# Modulating medial prefrontal cortex activity using real-time fMRI neurofeedback: Effects on reality monitoring performance and associated functional connectivity

**DOI:** 10.1016/j.neuroimage.2021.118640

**Published:** 2021-12-15

**Authors:** J.R. Garrison, F. Saviola, E. Morgenroth, H. Barker, M. Lührs, J.S. Simons, C. Fernyhough, P. Allen

**Affiliations:** a Department of Psychology, University of Cambridge, Downing St, Cambridge CB2 3EB, United Kingdom; b Behavioral and Clinical Neuroscience Institute, University of Cambridge, Downing St, Cambridge CB2 3EB, United Kingdom; c School of Psychology, University of Roehampton, Whitelands College, Holybourne Avenue, London SW15 4JD, United Kingdom; d CIMeC, Center for Mind/Brain Sciences, University of Trento, Rovereto, Trento 38068, Italy; e Institute of Bioengineering, École Polytechnique Fédérale de Lausanne (EPFL), Route Cantonale, Lausanne 1015, Switzerland; f Department of Radiology and Medical Informatics, University of Geneva, Geneva, Switzerland; g Department of Cognitive Neuroscience, Maastricht University, Maastricht 6200 MD, The Netherlands; h Research Department, Brain Innovation B.V., Oxfordlaan 55, Maastricht 6229 EV, The Netherlands; i Department of Psychology, Durham University, Upper Mountjoy, South Rd, Durham DH1 3LE, United Kingdom; j Department of Psychosis Studies, Institute of Psychiatry, Psychology and Neuroscience, Kings College London, De Crespigny Park, London SE5 8AF, United Kingdom; k Department of Psychiatry, Icahn Medical Institute, Mount Sinai Hospital, 1 Gustave L. Levy Place, Box 1230, New York, NY 10029, USA

## Abstract

Neuroimaging studies have found ‘reality monitoring’, our ability to distinguish internally generated experiences from those derived from the external world, to be associated with activity in the medial prefrontal cortex (mPFC) of the brain. Here we probe the functional underpinning of this ability using real-time fMRI neurofeedback to investigate the involvement of mPFC in recollection of the source of self-generated information. Thirty-nine healthy individuals underwent neurofeedback training in a between groups study receiving either Active feedback derived from the paracingulate region of the mPFC (21 subjects) or Sham feedback based on a similar level of randomised signal (18 subjects). Compared to those in the Sham group, participants receiving Active signal showed increased mPFC activity over the course of three real-time neurofeedback training runs undertaken in a single scanning session. Analysis of resting state functional connectivity associated with changes in reality monitoring accuracy following Active neurofeedback revealed increased connectivity between dorsolateral frontal regions of the fronto-parietal network (FPN) and the mPFC region of the default mode network (DMN), together with reduced connectivity within ventral regions of the FPN itself. However, only a trend effect was observed in the interaction of the recollection of the source of Imagined information compared with recognition memory between participants receiving Active and Sham neurofeedback, pre- and post- scanning. As such, these findings demonstrate that neurofeedback can be used to modulate mPFC activity and increase cooperation between the FPN and DMN, but the effects on reality monitoring performance are less clear.

## 1 Introduction

Reality monitoring refers to the cognitive processes used to distinguish internally generated experiences from those perceived in the external world (Johnson and Raye, 1981). Theory suggests that individuals make reality monitoring discriminations regarding the source of information based on the balance between internal and external cues, perhaps associated with spatial, temporal, sensory, and semantic detail, along with cognitive content (Source Monitoring Framework; Johnson and Raye, 1981). It is further suggested that there is overlap between the cognitive processes involved in the real-time discrimination of internally and externally generated information and those involved in memory recall (Woodward and Menon, 2014). As such, and given the difficulties of testing the discrimination of externally and internally generated information in real time, reality monitoring has been investigated empirically using memory experiments that manipulate the recollection of internally generated and externally derived source information. Such experiments have typically involved recollection of whether words or images were seen or imagined during an encoding task or distinguishing between word-pairs read aloud by the participant themselves vs. the experimenter (Brookwell et al., 2013; Simons et al., 2017).

Neuroimaging studies in healthy individuals have linked reality monitoring with functional activity within the medial prefrontal cortex (mPFC) (see Simons et al., 2017 for review), with the possibility of a causal link supported by a small navigated repetitive transcranial magnetic stimulation study (*N* = 11; Subramaniam et al., 2020). Such a relationship is consistent with evidence implicating the mPFC in recalling internal vs. external aspects of context (Simons et al., 2008; Turner et al., 2008), in making inferences about the mental states of others (Frith and Frith, 2003) and more broadly, in tasks involving self-referential judgements (Davey et al., 2016; Qin and Northoff, 2011; van der Meer et al., 2010). A morphological basis has also been established between the extent of cortical folding within the paracingulate sulcus (PCS) which lies within the mPFC, and reality monitoring accuracy in healthy individuals (Buda et al., 2011), with a possible functional explanation relating to the differential connectivity of the paracingulate region as part of large-scale brain functional networks including the default mode (DMN) and fronto-parietal (FPN) networks (Fornito et al., 2012; Garrison et al., 2015; Metzak et al., 2015).

While behavioral source monitoring testing has shown reality monitoring to be a highly variable ability among healthy individuals (Buda et al., 2011), it can be questioned whether such memory tasks adequately operationalise perceptual reality monitoring. Correlational evidence to support the use of source monitoring tasks to assess reality monitoring comes from the study of hallucinations in schizophrenia (Simons et al., 2017). When patients are tested and their results compared with those of healthy individuals they have been found to show reduced reality monitoring accuracy even when their item recognition memory is intact (Brunelin et al., 2006; Waters et al., 2004) suggesting a possible source monitoring deficit rather than a mnemonic one. The use of source monitoring tasks to assess reality monitoring has also revealed an association between patients’ experience of hallucinations and enhanced externalising bias (Simons et al., 2017) supporting the idea of a deficit in self recognition underlying hallucinations (Frith and Done, 1988). Furthermore, investigation of the reality monitoring impairment in schizophrenia suggests it is mediated by task specific mPFC dysfunction (Garrison et al., 2017a; Vinogradov et al., 2008) and we have also found a consistent morphological connection with the experience of hallucinations in patients associated with lower levels of cortical folding within the PCS (Garrison et al., 2015; Rollins et al., 2020). There is thus empirical evidence both for the use of source monitoring tasks in measuring reality monitoring, and for the role of the mPFC region in reality monitoring. Given these findings, attention has now turned to understanding the wider functional networks involved in the reality monitoring process.

In recent years a number of studies have used real-time fMRI neurofeedback (henceforth ‘fMRI neurofeedback’) to train individuals to self-regulate neural activity in brain regions and networks thought to underlie certain behaviors (deCharms et al., 2005), cognitive functions (Sherwood et al., 2016; Zhang et al., 2013a, 2013b) and psychiatric symptoms (Linden et al., 2012; Morgenroth et al., 2019; Orlov et al., 2018). Researchers are able to focus on, and to precisely define, target regions or networks of interest due to the high level of spatial resolution that can be achieved using MRI. As fMRI neurofeedback allows individuals to monitor and self-regulate their own brain activity in real time (Thibault et al., 2018), training individuals to regulate neural activity in particular brain regions or networks enables causal inferences to be made about that region's involvement in a certain behavior or function. Moreover, if a region or network is known to be involved in a pathological behavior, such as hallucinations, then altering activity in the region or network may have therapeutic benefits (Humpston et al., 2020).

The initial aim of our study was to determine whether healthy volunteers could be trained to self-regulate activity in their mPFC using fMRI neurofeedback during a single scanner visit. We then examined the effects of the fMRI neurofeedback training on reality monitoring accuracy for self-generated information (‘Imagined items’) using an established and validated behavioral reality monitoring task offline (Garrison et al., 2017b). We also used exploratory Independent Component Analysis (ICA) to examine whether neurofeedback training targeting the mPFC altered resting state functional connectivity (rsFC) associated with reality monitoring ability for Imagined items.

Our focus was on the PCS region of the mPFC and we therefore concentrated our ICA on components that correlated with two labeled functional networks which both incorporate regions of the PCS and have previously been implicated in reality monitoring (Fornito et al., 2012; Metzak et al., 2015). The DMN (Raichle, 2015; Yeo et al., 2011) includes areas of ventral medial and dorsomedial PFC (including anterior paracingulate cortex), posterior cingulate cortex / precuneus, and lateral parietal cortices (Raichle, 2015; Yeo et al., 2011). The DMN is observed to be more active when the brain is at rest and during internally directed and self-referential processing (Qin and Northoff, 2011; Davey et al., 2016), but shows deactivations during tasks that require external attention (Harrison et al., 2008; Shulman et al., 1997). In contrast the FPN includes more posterior regions of paracingulate cortex and precuneus, lateral prefrontal cortices (especially middle frontal gyrus), anterior inferior parietal lobule, anterior insula and subcortical structures including the caudate and thalamus (Uddin et al., 2019; Yeo et al., 2011). The FPN is a component of the brain's External Attention System which shows increased activity during cognitive tasks (Marek and Dosenbach, 2018). Indeed it is suggested that there is a fundamental functional distinction between the DMN and External Attention System (Golland et al., 2008) as the two networks often show antagonistic and anti-correlated activity depending on the internal or external nature of task demands (Hugdahl et al., 2019; Marek and Dosenbach, 2018; Spreng et al., 2013). The FPN is specifically implicated in attentional aspects of cognitive control by flexibly coupling with either the DMN or Dorsal Attention component of the External Attention System depending on internal / external task demands (Marek and Dosenbach, 2018).

The DMN has been implicated in reality monitoring processing, consistent with the internal attentional demands of the task: Metzak et al., 2015 showed that deactivation observed within the DMN during a non self-referential source monitoring task (consistent with external attention) was diminished during a reality monitoring task leading to higher net levels of network activity. However, reality monitoring is also known to coactivate areas of both the DMN and External Attention System including the FPN (Simons et al., 2008) consistent with internal and external task demands. Fornito et al. (2012) used fMRI to provide more nuanced insight into the network interactions during reality monitoring finding that increased cooperation between the DMN and right lateralised FPN component of the External Attention System was associated with more rapid reality monitoring memory recollection. Furthermore, this cooperation was facilitated by a component of the DMN, with the right posterior cingulate cortex in particular appearing to act as an information processing hub to provoke context dependent reconfigurations from cooperative to antagonistic dynamics between the networks.

Our hypotheses were as follows. Firstly, that relative to a Sham neurofeedback condition, participants receiving veridical Active neurofeedback would show an interaction for increased mPFC activity (or reduced deactivation) over the course of three runs of neurofeedback training targeting the mPFC / PCS. Secondly, given our previous finding that lower reality monitoring accuracy in patients with schizophrenia was associated with reduced mPFC activity (Garrison et al., 2017a), that up-regulation of activity in the mPFC brought about by fMRI neurofeedback training would be associated with improvement in reality monitoring accuracy for the recollection of Imagined items, but not with general item recognition. Finally, and consistent with the research (Fornito et al., 2012; Metzak et al., 2015) discussed above, participants receiving Active neurofeedback relative to Sham, would show increased rsFC (post > pre neurofeedback training) associated with reality monitoring for Imagined items in areas of the DMN (consistent with enhanced self-recognition) and FPN, with evidence of increased rsFC cooperation between the DMN and FPN (both supporting a shift towards internal attention).

## 2 Methods

### 2.1 Participants

39 healthy individuals (males = 15) participated in a between-groups, single blind randomised control design. Participants were recruited from the University of Roehampton, Royal Holloway University of London and from the general public using adverts on social media. The mean age of participants was 21.9 years (SD = 3.0 years) and there were 36 right handed and 3 left handed participants. Participants had no prior neurological or medical illness and were not using any psychiatric medication.

21 participants were randomly assigned to the Active neurofeedback condition targeting the mPFC, and 18 participants to the Sham neurofeedback condition. Our group sample sizes were supported by a review of published fMRI neurofeedback studies where moderate to strong effect sizes were achieved using behavioral or clinical measures similar to those used in the current study, with group sample sizes ranging from 7 to 30 (mean = 13.6, SD = 6.6; Bauer et al., 2020; deCharms et al., 2005; Pamplona et al., 2020; Sherwood et al., 2016; Young et al., 2014; Zhang et al., 2013a, 2013b). In these studies, correlation coefficients between activity increases due to neurofeedback and associated behavioral or clinical effects ranged from 0.37 to 0.96 (mean = 0.65, SD =0.28), and group differences between Active and Sham groups ranged from *d* = 0.57 to *d* = 0.78 (mean = 0.66, SD = 0.10). Based on these mean prior effect sizes, our samples gave an estimated 96% power to detect a possible correlation effect, and 65% to detect a possible group difference effect (alpha = 0.05, 1 tailed).

There were no significant differences between the groups (Active vs. Sham) in terms of age [t(37) = 1.154, *p* = .539], sex [χ^2^ (1) = 0.003, *p* = .959] or handedness [χ^2^ (1) = 2.786, *p* = .235].

### 2.2 Ethics statement

The study was approved by the University of Roehampton Ethics Committee and all participants gave written informed consent prior to taking part in the study.

### 2.3 Study protocol

**Fig. 1:**
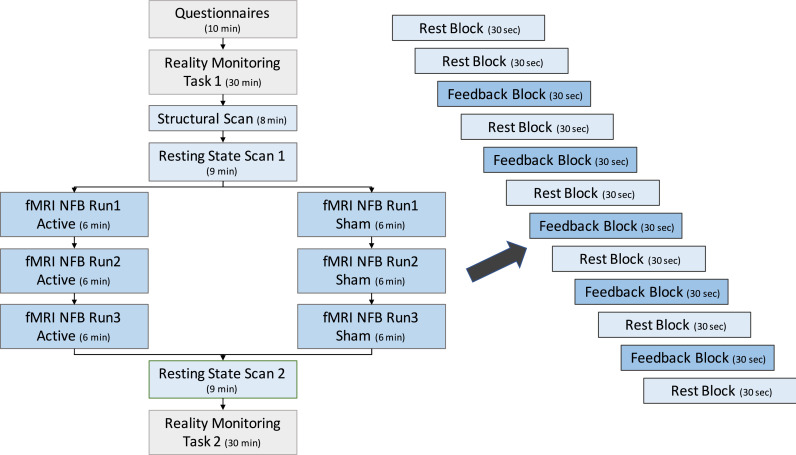
Study Protocol. Participants were randomly allocated to Active and Sham groups but aside from neurofeedback, experienced identical protocols. Blue text-boxes indicate scanned sessions, grey boxes indicate off-line activities. Note: NFB = neurofeedback. An extra rest session is included at the start of the imaging run to establish a longer baseline for the neurofeedback contrast. (For interpretation of the references to color in this figure legend, the reader is referred to the web version of this article.)

Participants underwent questionnaire assessment, offline reality monitoring testing (pre and post neurofeedback) and scanning in a single three-hour visit. Details of the study protocol are given in Fig. 1.

### 2.4 Assessment for schizotypy and proneness to hallucinations

Individuals’ proneness to hallucinations and schizotypy were assessed by self-report using a written questionnaire prior to scanning, to ensure that the groups were matched on trait measures previously related to reality monitoring and hallucinations (Simons et al., 2017). Hallucination proneness was assessed using (Morrison et al., 2000) Revised Launay-Slade Hallucination Scale (Bentall and Slade, 1985). The LSHS-R scale comprises twelve questions with each item scored on a five-point Likert scale ranging from ‘never’ (0) to ‘almost always’ (4). Total scores could thus range from 0 to 60 with higher scores indicating a greater predisposition to hallucinations. The mean LSHS-R score of the Active group was 16.3 (SD = 6.6) and for the Sham group 13.6 (SD = 7.2), there was no significant difference between the groups [*t*(37) = 1.206, *p* = .235].

Schizotypy was assessed using the Brief O-LIFE scale (OLIFE-B), a 30-item shortened version of the original 104-item Oxford-Liverpool Inventory of Feelings and Experiences (Mason et al., 2005, 1995). OLIFE-B comprises two sub-scales of 15 items each. Odd numbered items contribute to OLIFE-B(-) which is a measure of introvertive anhedonia (lack of enjoyment and social withdrawal), a key negative feature of schizotypy, and even numbered items to OLIFE-B(+) as a measure of unusual experiences / positive features. Each item is scored with a simple 1 for ‘yes’ and 0 for ‘no’ with the scores summed for each scale to give a value from 0 to 15. The mean OLIFE-B(-) score for the Active group was 3.3 (SD = 2.9) compared with 2.7 (SD = 2.5) for the Sham group, while the mean OLIFE-B(+) score for the NFB group was 6.1 (SD = 4.0) compared with 5.3 (SD = 2.8) for the Sham group. There was no significant difference between the groups for either OLIFE-B(-) [t(37) = 0.695, *p* = .491], or OLIFE-B(+) [t(37) = 0.722, *p* = .475].

### 2.5 MRI acquisition

All MRI scans were acquired on a 3 Tesla Siemens Magnetom TIM Trio scanner using a 32-channel head coil at the Combined Universities Brain Imaging Center at Royal Holloway, University of London (CUBIC; http://www.cubic.rhul.ac.uk). Each participant underwent an anatomical scan which comprised a T1-weighted Magnetization Prepared Rapid Acquisition Gradient Echo (MPRAGE) image (1mm^3^ resolution, in plane resolution 256 × 256 × 176 slices, acquisition time approximately 5 min). fMRI neurofeedback runs were also acquired for each participant comprising of five feedback vs. seven rest blocks each lasting 30 s (Fig. 1). Resting state fMRI scans were collected from all participants before and after the acquisition of the 3 x fMRI neurofeedback training runs. All functional resting state and neurofeedback scans were acquired using echo-planar image sequences: TR = 2 s, TE = 40 ms, 28 slices, 4 mm slice thickness, in-plane resolution 3 mm × 3 mm.

### 2.6 Anatomical localiser

The mPFC target region for fMRI neurofeedback was delineated anatomically in all participants using their T1-weighted anatomical scan. To aid localization we delineated an anatomical region along the bilateral PCS because the morphology of this sulcus lies within the mPFC and has been shown to be associated with reality monitoring performance (Buda et al., 2011). The bilateral PCS was delineated manually using tools in Turbo-BrainVoyager (Brain Innovation, Maastricht, Netherlands) by an investigator trained to recognise the morphology and anatomy of the region (JG). The extent of the mean binary mask across all participants is shown in Fig. 3A. The bilateral PCS target regions were then transferred into a Turbo-BrainVoyager file format and used to define the neurofeedback target region (volume of interest) on echo-planar images during neurofeedback training runs.

### 2.7 fMRI neurofeedback

Neurofeedback was administered over three x 6 min scanner runs during a single scanner visit using Turbo-BrainVoyager with each run composed of Feedback and Rest blocks (see Fig. 1). Reconstructed DICOM images were directly transferred from the MRI scanner via a secure data transfer protocol to an analysis computer where TBV was installed. Pre-processing was performed on all transferred images, including Gaussian spatial smoothing with a kernel of 4 mm full width half maximum, and motion correction. The functional data was registered to the anatomical scan acquired at the beginning of the scanning session.

Participants received either Active (based on the fMRI neurofeedback signal from the PCS) or Sham signal during Feedback blocks via a visual ‘gauge’ interface (Fig. 3C). Participants were instructed to move the gauge ’up’ during Feedback blocks, so that all cells in the gauge were turned grey (achieved in active blocks by up-regulation of the BOLD signal in the PCS). Participants were instructed to relax during Rest blocks. No specific direction or instructions were given to participants regarding how to self-regulate their neurofeedback signal but participants were told to allow 5 to 7 s for their efforts to result in a change in the gauge (to allow for the haemodynamic response). During Feedback blocks, a continuous signal from the PCS target area was displayed via the visual gauge and updated for every scan volume (TR = 2 s). Changes in amplitude were indicated in terms of the percentage signal change, calculated as the current signal value compared with the average value determined from the immediately preceding rest block (Turbo-BrainVoyager User's Guide). The thermometer was scaled with a maximum value of 0.5%, and gradations of 0.05%, chosen to match previous successful neurofeedback studies (Cohen Kadosh et al., 2016; Linden et al., 2012). The thermometer remained visible during rest blocks, with a change in colour of the top box on the thermometer gauge from red to white indicating a switch from a Rest to a Feedback block, simultaneously accompanied by a two second presentation of the word ‘*Rest’* (and vice versa with the words ‘*Move Thermometer’*). The Active feedback signal was calculated using a real-time general linear model based on a single predictor for the Feedback / Rest onsets function convolved with the haemodynamic reference function, with the top third of the voxels in the target PCS region (defined by the t value for the contrast of predictor vs. baseline) used to compute the signal. The Sham feedback signal was based on a saved pattern of randomised activity at a similar level of intensity to active feedback (provided by Turbo-BrainVoyager technical support), but derived from no specific brain region.

### 2.8 Offline fMRI data analyses

Functional data were analyzed using SPM12 (http://www.fil.ion.ucl.ac.uk/spm). Functional volumes were spatially realigned to the first image of the first series and volumes normalised against the MNI reference brain using tri-linear interpolation, and smoothed with an isotropic default 8 mm full-width half-maximum Gaussian kernel. Block analysis was undertaken with separate regressors coding for the onsets of Feedback and Rest blocks. These together with the six regressors coding head motion parameters, comprised the full model for each run. The data and model were high-pass filtered to a cut off of 1/ 128 Hz.

A simple contrasts of interest analysis was performed on individual participant data at the first level using the contrast of Feedback > Rest. Each participant's contrast file was then submitted to a full factorial ANOVA at the second level to test the interaction between group (Active vs. Sham) and neurofeedback training run (Run1, Run2, Run3). To test our first *a priori* hypothesis, we examined the interaction term within the mPFC region using a small volume correction (SVC) for multiple comparisons, with a familywise-error (FWE) corrected voxel-wise height threshold of *p* < .05. The region of interest was defined as an 8 mm radius sphere (consistent with the smoothing kernel used for pre-processing) centered on *a priori* coordinates of a PFC brain region which is dysfunctional in schizophrenia [15, 52, −1; Whalley et al., 2004] and associated with reality monitoring in healthy subjects (Simons et al., 2006). Significant effects were reported at a FWE corrected voxel level of *p* < .05.

### 2.9 Reality monitoring task

**Fig. 2:**
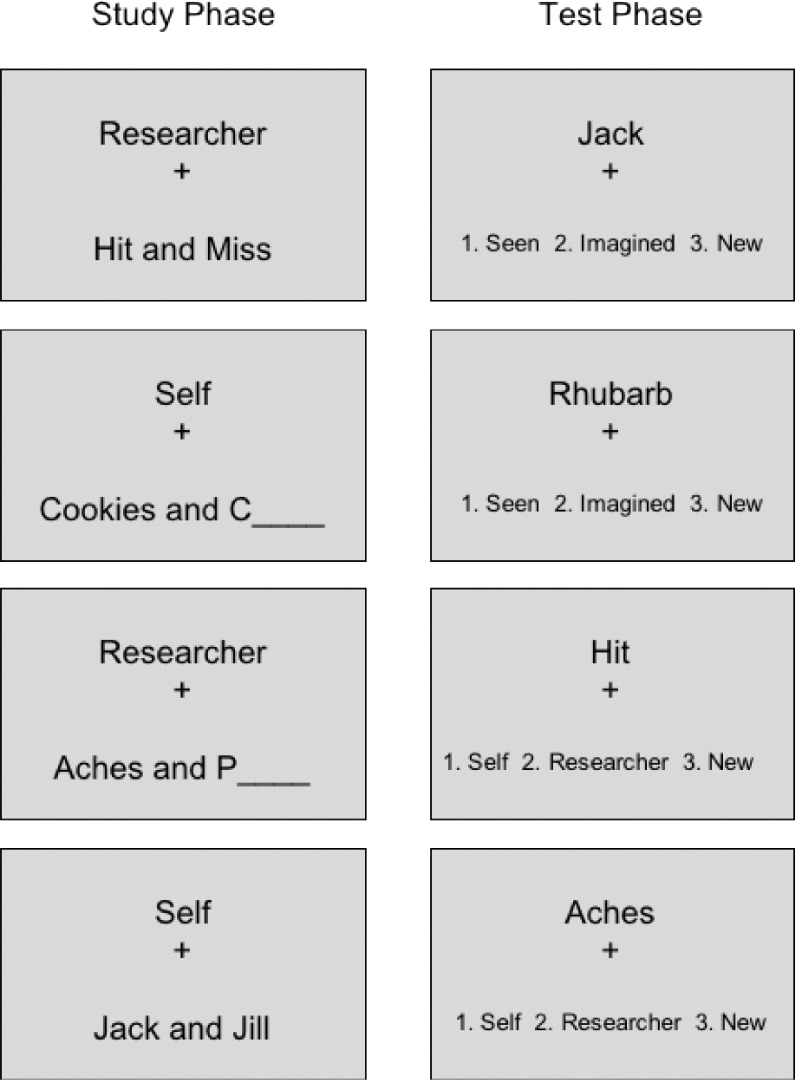
Stimuli used in the study and test Phases of the Reality Monitoring Tasks. Note: In a 2 × 2 design, either the participant or researcher spoke aloud the stimuli, which were presented either complete (Seen) or incomplete (requiring the second word to be Imagined). Subjects were then presented at test with the first word of a word-pair, and asked to respond as to whether the accompanying word had been Seen or Imagined, or if the presented word was New; or whether they or researcher had read aloud the word-pair, or the presented word was New.

Two reality monitoring tasks were used, one prior to scanning and one after scanning was complete – these were identical apart from the choice of word-pair stimuli which were unique to each version. The task used was similar to that described (Garrison et al., 2017b) involving a series of five blocks each lasting around 5 min, with each block comprising a study phase when a series of 24 word-pairs were presented and a test phase (Fig. 2). In the test phase, the participant was asked whether a word had previously been presented during the study phase within an intact word-pair using the response ‘*Seen’*, or had been presented in a word-pair which had needed to be completed by imagining the missing word, with the response ‘*Imagined’*. Participants were also required to judge whether a word-pair had previously been spoken aloud by themselves (‘*Self’* response) or was spoken by the researcher (‘*Researcher’* response). 12 previously unstudied words were used in addition to the 24 word-pairs from the study phase for each test phase, requiring a ‘*New’* response. The stimuli comprised 360 well-known word-pairs across the two tasks (e.g. ‘*Hit and Miss’, ‘Rhubarb and Custard*’) which were pilot tested before the study to ensure familiarity among adults in the target demographic range. Six word-pairs were presented in four combinations of Self / Researcher x Seen / Imagined for each study phase. A practice task was used before testing to ensure participants’ familiarity and understanding of the task protocol.

Each study trial commenced with a screen indicating whether the participant or researcher should read aloud the word-pair. The word-pair was then shown, either complete (Seen trials) or with only the first letter of the second word provided such that the second word needed to be self-generated (Imagined trials). In both cases the participant or researcher then had 3 s to read aloud the word-pair, completing it as necessary for Imagined trials. Each study phase was followed by its corresponding test phase, consisting of one sub-block for each of the two reality monitoring conditions. The sub-blocks commenced with a question screen indicating which condition was being tested, i.e. for the Seen / Imagined condition: ‘*Was the accompanying word Seen or Imagined or New?*’, and for the Subject / Researcher condition: ‘*Was the accompanying word said by Self or Researcher or New?*’ These were then followed by a test screen containing the first word from one of the studied word-pairs, or a new word, together with the instruction to provide the appropriate response. Participants had four seconds in which to respond. The order of presentation of sub-blocks in the test phase alternated across the five full blocks of the task and was counterbalanced across participants. The word-pairs assigned to the Seen / Imagined and Self / Researcher conditions, as well as New words, were also counterbalanced across participants.

### 2.10 Analysis of behavioral data

Old / New recognition accuracy was calculated as the adjusted item recognition score (hits minus false alarms) in order to differentiate sensitivity from response bias. Hits were defined as the proportion of words correctly recognised as previously seen during the study phase and false alarms as the proportion of new words incorrectly endorsed as old. To exclude the effects of changes in recognition memory from the reality monitoring assessment, reality monitoring accuracy was calculated as the number of accurate source responses divided by the number of correct responses recognising an item as old. Reality monitoring and recognition memory accuracy were assessed separately for Seen / Imagined sub-blocks, and for Self / Researcher sub-blocks.

We focused our behavioral analysis on changes in recognition memory accuracy and reality monitoring accuracy for Imagined items and carried out planned contrasts of accuracy scores post-scanning with scores pre-scanning for the two groups separately. To assess the specificity of any group effect to self-recognition we then analyzed this behavioral data using a 3-way mixed ANOVA with group (Active vs. Sham) as a between subjects factor, and session (before or after scanning), and condition (recognition memory or reality monitoring accuracy for Imagined items) as within subjects factors.

To investigate a possible direct association between improved self recognition ability and increased functional activity within mPFC, we carried out a correlation analysis between the change in memory accuracy scores for Imagined items, and for recognition memory as a control, with the signal change in the peak PFC voxel between training Run1 and Run3.

### 2.11 Resting state functional connectivity (rsFC) analyses

Resting state data (acquired pre and post fMRI neurofeedback) were analyzed with FMRIB Software Library (FSL; Jenkinson et al., 2012). Volume reorientation and head motion correction was performed using MCFLIRT software (Jenkinson et al., 2002) with the rigid body transformation default setting. Brain extraction was undertaken on both the T1-weighted images and EPI motion corrected sequence scans using BET (Smith, 2002) with the *f* parameter set to 0.5, and with visual inspection of the images to ensure appropriate extraction of the brain. Spatial smoothing was applied using a default 6 mm full-width half maximum Gaussian kernel (twice the voxel size of the images; Worsley and Friston, 1995), with a band-pass filter [0.01–0.1 Hz] cut-off. Co-registration and normalization to standard MNI template were undertaken using FLIRT software (Jenkinson et al., 2002). This involved three steps: (i) co-registration of the mean standard functional image to the T1-weighted brain extracted image, (ii) saving the transformation between the T1 anatomical images to MNI space (trilinear interpolation), (iii) application of this transformation to the pre-filtered functional sequence.

The pre-processed resting-state fMRI data was then analyzed using Multivariate Exploratory Linear Optimized Decomposition into Independent Components 3.0 (MELODIC). The multiple 4D data sets were decomposed into their distinct spatial and temporal components using Independent Component Analysis (ICA). As the aim of the analysis was to compare group differences post > pre fMRI neurofeedback training, we did not assume consistent temporal responses between subjects. As such, the ICA was temporarily concatenated (FSL; Jenkinson et al., 2012). A single 2D analytical run was undertaken on the concatenated data matrix obtained by stacking the 2D data matrices of every dataset for all subjects in the group. The Independent Component number was manually set to 20 (Abou Elseoud et al., 2011, 2010; Calhoun et al., 2004; Li et al., 2007).

In order to separate noise components from the underlying resting-state networks, two Independent Components used in the functional connectivity analysis were chosen after establishing a threshold of *r*-value > 0.2 of correlation as recommended in FSL (Jenkinson et al., 2012) with the DMN and FPN reference networks (Yeo et al., 2011). The actual correlations achieved were well in excess of this threshold and together with the Independent Components used and their comparison to the reference networks, are shown in Supplementary Fig. S1.

The two Independent Components were then submitted to second level analysis. Pre-training spatial maps for each subject were first contrasted with post-training maps (i.e. post > pre fMRI neurofeedback). A dual regression comprising group-average ICA analysis followed by single subject estimation of specific group-level spatial maps (Beckmann et al., 2009; Nickerson et al., 2017) was then performed to investigate group (Active vs. Sham) differences in rsFC related to the fMRI - neurofeedback training. To restrict the rsFC analysis to regions associated with changes in reality monitoring accuracy for Imagined items, the Z-scores for the change in accuracy for the recollection of Imagined items (post > pre fMRI neurofeedback, calculated as (difference score – group mean) / standard deviation) were added as a variable of interest to the general linear models to investigate group effects on rsFC (post > pre fMRI neurofeedback). Statistical group differences were tested using non-parametric permutation testing, with threshold-free cluster enhancement (Smith and Nichols, 2009). Functional connectivity results are reported for *p* < .05 FWE threshold corrected for multiple comparisons across voxels, and *p* < .025 with Bonferroni correction for multiple comparisons across the two Independent Components (networks).

### 2.12 Data and software availability

Data obtained in the study has been made publicly available: https://doi.org/10.17863/CAM.76750. The software used in the study is publicly available with sources cited in the manuscript.

## 3 Results

### 3.1 fMRI neurofeedback

**Fig. 3:**
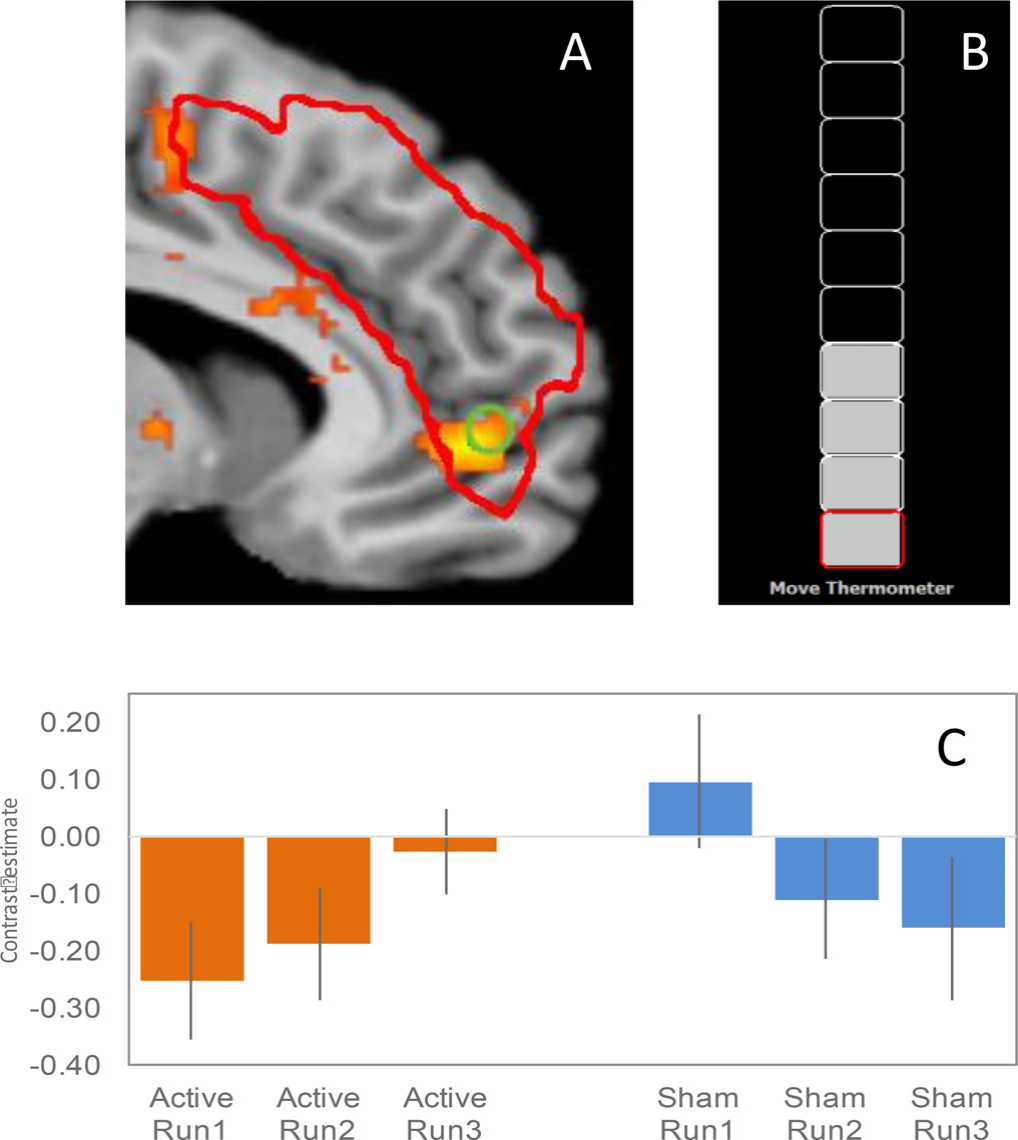
A. Statistical parametric map showing group (Active vs. Sham) by fMRI neurofeedback training run (interaction effect) in the mPFC region of interest (green circle). The red boundary line shows the mean PCS mask across all 39 participants. Images are centered on the peak mPFC voxel at [8, 48, −4, *p* = .045 FWE], and activity thresholded at *p <* 〈 .05 uncorrected for visualisation presentation. B. Example of the visual neurofeedback gauge interface. C. Plot from the peak mPFC voxel [8, 48, 4] showing increasing activation / reducing deactivation for the Feedback >〉 Rest contrast across the three neurofeedback runs in the Active group, and decreasing activition / increasing deactivation in the Sham group. Note: The mean number of 1mm^3^ voxels in the anatomical localizer for participants in the Active group was 12,961 (SD = 3,249) compared to 12,885 (SD = 2,104) for participants in the Sham group [*t*(37) = 0.085, *p* = .932]. (For interpretation of the references to color in this figure legend, the reader is referred to the web version of this article).

Analysis of the fMRI data over the three neurofeedback scanning runs revealed a significant group (Active vs. Sham) x run (Run1, Run2, Run3) interaction in the mPFC region of interest [peak 8, 48, −4, *Z* = 2.79, p_FWE peak_ = 0.045, SVC; Fig. 3A].

Violin plots showing the distribution of participants’ fMRI contrast estimate from the peak mPFC voxel [8, 48, −4] reveal a high level of individual differences in values across the two groups (Supplementary Fig. S2).

### 3.2 Reality monitoring

**Fig. 4:**
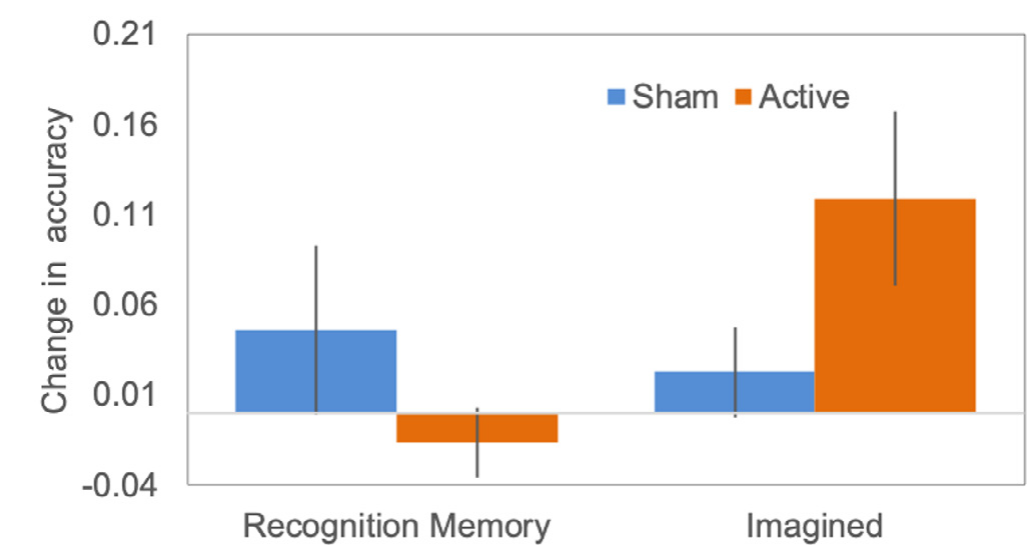
Changes in recognition memory and reality monitoring accuracy for Imagined items (post > pre fMRI neurofeedback training) for participants within the Active (orange) and Sham (blue) groups. Violin plots showing the group distributions of this data are given in Supplementary Fig. S3.

Planned contrasts of recognition memory and reality monitoring accuracy for Imagined items pre and post scanning revealed a significant increase in reality monitoring for Imagined items post scanning in Active group participants but not in Sham group participants (Table 1 and Fig. 4). This effect was not significant following Bonferroni correction for multiple comparison. There were no significant differences in recognition memory accuracy post scanning in either group.

**Table 1:** Accuracy for recognition memory and reality monitoring for Imagined items in Active and Sham group participants, pre and post fMRI neurofeedback scanning.

	Recognition Memory		Reality Monitoring for Imagined Items	
	Active	Sham	Active	Sham
Before Scanning	.737 (.166)	.771 (.189)	.683 (.266)	.707 (.314)
After Scanning	.719 (.180)	.817 (.075)	.800 (.172)	.736 (.324)
*t*-test	t(20) = - 0.930 *p* = .364	t(17) = 0.991 *p* = .336	t(20) = 2.422 *p* = .025	t(17) = 0.946 *p* = .357

Notes: SD in parentheses; none of the *t*-test results are significant after Bonferroni correction for multiple comparisons for four tests carried out.

To compare the effect of active neurofeedback on reality monitoring accuracy for Imagined items with that for recognition memory (Fig. 4), we then analyzed the behavioral data using a 3-way mixed ANOVA with group (Active vs. Sham) as a between subjects factor and session (before or after scanning), and condition (recognition memory or reality monitoring for Imagined Items) as within subjects factors. This revealed no main effects of group F(37,1) = 0.154, *p* = .697, η_p_^2^ = 0.004 or memory condition F(37,1) = 0.636, *p* = .430, η_p_^2^ = 0.017, but a significant effect of session F(37,1) = 5.928, *p* = .020, η_p_^2^ = 0.138. There were no significant two way interactions, but there was a trend effect in the three way interaction of group x session x memory condition, F(37,1) = 3.976, *p* = .054, η_p_^2^ = 0.097.

There were no significant correlations between the signal change in the peak mPFC voxel [8, 48, -4] between training Run1 and Run3, and the change in reality monitoring accuracy for Imagined items in either the Active (*r* = 0.161, *p* = .485) or Sham group (*r* = 0.093, *p* = .713), nor with the change in recognition accuracy in either the Active (*r* = 0.086, *p* = .710) or Sham group (*r* = 0.095, *p* = .707).

### 3.3 Resting state functional connectivity

**Fig. 5:**
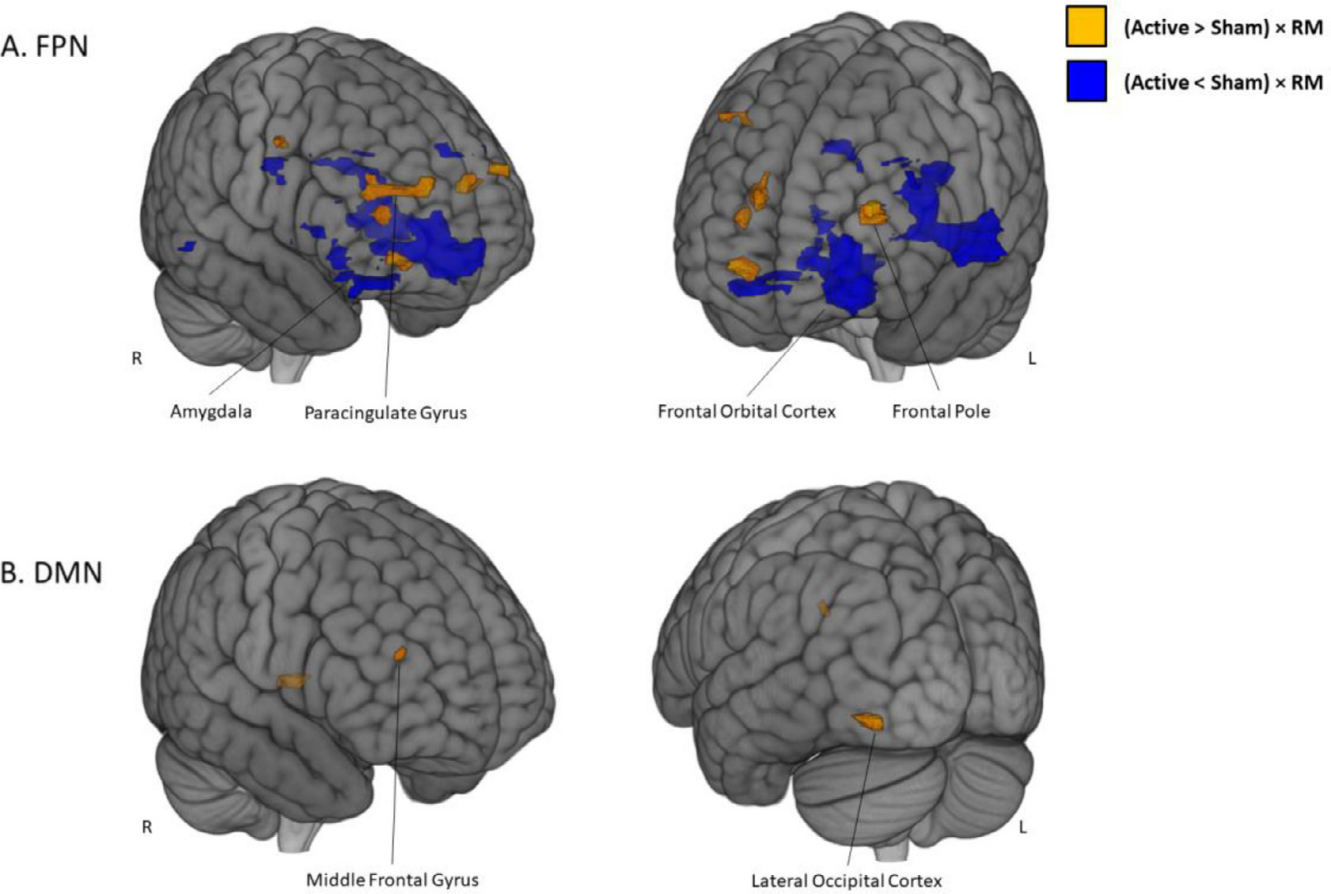
3D brain rendering showing areas of increased (yellow) and decreased (dark blue) resting state functional connectivity due to Active compared to Sham fMRI neurofeedback, and associated with increases in reality monitoring accuracy for Imagined items. A: FPN Independent Component, B: DMN Independent Component. Significant ICA results are shown for *p-v*alue < 0.05 FWE voxel-wise corrected and *p*-value < .025 Bonferroni corrected for two Independent Components tested. Scatter plots of participant data are given in Supplementary Fig. S6 (For interpretation of the references to color in this figure legend, the reader is referred to the web version of this article.)

Results of the Independent Component interaction analysis showing group differences in pre vs. post changes in rsFC following neurofeedback training and associated with changes in reality monitoring accuracy for Imagined items are shown in Fig. 5 and Tables 2 and 3.

**Table 2:** Group differences in changes in resting state functional connectivity following fMRI neurofeedback scanning associated with changes in reality monitoring accuracy for Imagined items, within the DMN Independent Component.

Contrast	Cluster	Cohen's d	*t*-value	*p*-value	MNI coordinates			Extent of coverage
					x	y	*z*	
Active > Sham	1	1.9	6.1	.010	−38	−82	−8	Lateral Occipital Cortex, Fusiform Gyrus
	2	1.8	5.6	.016	29	38	20	Middle Frontal Gyrus

**Note**: Significant results are shown for *p*-value < 0.05 FWE voxel-wise corrected and *p*-value < 0.025 Bonferroni corrected for multiple Independent Components tested. Note: The results in Tables 2 and 3 are displayed against overlays of the standard DFM and FPN in Supplementary Figs. S4 & S5 to highlight possible cooperation between these networks associated with increased attention to internally focused tasks.

**Table 3:** Group differences in changes in resting state functional connectivity following fMRI neurofeedback scanning associated with changes in reality monitoring accuracy for Imagined items, within the FPN Independent Component.

Contrast	Cluster	Cohen's d	*t*-value	*p*-value	MNI coordinates			Extent of coverage
					*x*	*y*	z	
Active > Sham	1	1.6	5.2	.011	14	46	24	Paracingulate Gyrus
	2	2.0	6.2	.008	−34	46	28	Superior Frontal Gyrus
	3	1.9	5.9	.010	26	42	−8	Middle Frontal Gyrus
	4	2.2	6.9	.005	−30	34	20	Middle Frontal Gyrus, Inferior Frontal Gyrus, pars triangularis
	6	1.8	5.7	.017	54	−2	44	Middle Frontal Gyrus, Precentral Gyrus, Postcentral Gyrus
	7	1.7	5.5	.022	−46	46	16	Middle Frontal Gyrus
Sham > Active	2	1.4	4.5	.015	10	26	−16	Middle Frontal Gyrus
	4	1.5	4.7	.015	14	−2	−12	Amygdala, Pallidum
	5	1.7	5.3	.014	−18	−18	28	Caudate
	6	1.5	4.6	.014	30	30	−20	Inferior Frontal Gyrus
	7	1.6	1.2	.010	−30	−18	12	Putamen
	9	1.9	6.1	.001	−18	34	−20	Inferior Frontal Gyrus
	10	1.6	4.9	.021	−38	14	28	Middle Frontal Gyrus, Inferior Frontal Gyrus, pars opercularis, Precentral Gyrus
	11	1.3	4.1	.023	−2	−2	32	Cingulate Gyrus
	12	1.6	5.2	.024	38	−70	−12	Fusiform Gyrus, Lateral Occipital Cortex
	13	1.0	3.1	.017	−30	−70	−8	Fusiform Gyrus, Lateral Occipital Cortex,
	14	1.4	4.4	.023	−6	−26	8	Thalamus
	15	2.0	6.4	.021	26	−62	68	Lateral Occipital Cortex, Superior Parietal Lobule
	16	0.8	2.3	.024	−38	−10	48	Precentral Gyrus

Within the DMN Independent Component, fMRI neurofeedback increased rsFC relating to reality monitoring for Imagined items in the right middle frontal gyrus and left fusiform gyrus. There were no regions where fMRI neurofeedback decreased rsFC (Fig. 5 and Table 2). The middle frontal gyrus cluster overlapped with the standard FPN (Supplementary Fig. S5).

Within the FPN Independent Component, Active fMRI neurofeedback increased rsFC within more dorsal regions of the lateral and medial prefrontal cortex, including the right paracingulate gyrus (which overlapped with the standard DMN, Supplementary Fig. S4), the left superior and inferior frontal gyrus and bilateral middle frontal gyrus. Decreased rsFC was also seen in more ventral prefrontal regions in the right middle frontal gyrus and the bilateral inferior frontal gyrus (extending to the left superior temporal gyrus), as well as in subcortical regions including left basal ganglia, amygdala and the left thalamus and in the bilateral occipital cortex and right superior parietal lobule (Fig. 5 and Table 3).

## 4 Discussion

In this study we have shown that participants provided with Active fMRI neurofeedback were able to successfully up-regulate activity within the mPFC over the course of three neurofeedback training runs undertaken in a single scanning session compared to participants receiving Sham neurofeedback training. These findings suggest that participants can learn to self-regulate and increase activity within their mPFC using the real-time fMRI neurofeedback protocol. We found only a trend effect in the behavioral analysis of pre vs. post neurofeedback reality monitoring accuracy for recollection of Imagined items. As such these findings do not provide conclusive evidence of a specific causal link between neurofeedback-induced changes in mPFC activity and reality monitoring ability. However, we did find significant rsFC group effects in regions associated with reality monitoring accuracy for Imagined items. This provides preliminary evidence of a possible link between Active neurofeedback training and changes in the networks underlying reality monitoring ability for Imagined items which may be mediated by mPFC activity.

The increase in functional activity following neurofeedback training in the Active group was measured in an mPFC region of interest based on a brain area previously shown to be associated with reality monitoring accuracy in healthy individuals and dysfunctional in schizophrenia (Simons et al., 2006; Whalley et al., 2004). Peak activity was observed in a voxel [8, 48, -4] that lay within the mean anatomical mask from all 39 participants. Inspection of the mean group functional activity pattern within the peak voxel during scanning (activity measured during feedback blocks relative to rest blocks), showed sequentially reduced deactivation in the Active group across the three feedback runs with sequentially increased deactivation in the Sham group (Fig. 3C).

Notably, violin plots of participants’ peak voxel activity indicated high individual variation across the two groups (Supplementary Fig. S2). This may help explain the observation of a mean level of deactivation in Run 1 for participants in the Active group compared to a mean level of activation in Run 1 from those in the Sham group, especially as to cause the thermometer to rise, participants needed to increase activity within the PCS ROI during a feedback block relative to the preceding rest block. Participants are likely to have employed a number of different strategies in their attempts to move the visual thermometer, some of which are likely to have been successful and some not. As the anterior mPFC is a key component of the DMN where deactivation is seen during external attention-demanding and non-self-referential tasks (Raichle, 2015; Spreng et al., 2013) we would expect these strategies to result in greater deactivation or activation within the mPFC ROI relative to the rest condition, depending on the extent of external and self-referential processing. Thus strategies involving greater self-referential processing might be expected to increase mPFC activity compared to the rest condition, whiles strategies utilising more external attention might be expected to decrease mPFC activity relative to the rest condition. When viewed on a within subject basis, the observed pattern of sequentially reduced deactivation over the three neurofeedback runs in participants in the Active group is consistent with a pattern of increasing success on the task, and suggests a net increase in internally directed attention required for effective self-regulation, while the pattern of increased deactivation over the three runs in the Sham group, particularly runs 2 and 3, is consistent with participants’ increased external focus as they failed to gain control over their ability to move the thermometer.

Overall, these results suggest that neural activity within the mPFC can be self-regulated in healthy volunteers and is consistent with previous fMRI neurofeedback studies that show that individuals can be trained to regulate activity in medial cortical regions such as the cingulate cortex (Mathiak et al., 2015; Zilverstand et al., 2017) and the precuneus (Zhang et al., 2013a, 2013b; Garrison et al., 2013).

We also observed alterations in the functional networks associated with changes in reality monitoring for Imagined items. Active neurofeedback targeting the mPFC was associated with increased rsFC within both the FPN and DMN Independent Components, primarily in dorsal frontal areas including paracingulate cortex. Active neurofeedback was also associated with reduced rsFC within the FPN network Independent Component in more ventral frontal regions, in subcortical areas (including thalamus and caudate) as well as areas of lateral parietal and occipital cortex. It thus appears that the effect of the Active neurofeedback may have been to increase connectivity between the dorsolateral frontal areas of the FPN (particularly middle frontal gyri, observed within both the FPN and DMN Independent Components) and the mPFC region of the DMN (paracingulate gyrus; see Supplementary Figs. S4 and S5), while also reducing connectivity within the FPN itself (across ventral lateral frontal regions and subcortical areas) and possibly also with sensory regions of the visual network. Although a speculative interpretation, this would be consistent with the effect of increased cooperation between the FPN and DMN (Fox et al., 2005; Hugdahl et al., 2019) as attention is switched more internally as Active group participants learn how to regulate mPFC activity during the neurofeedback task. This could then have a possible impact on behavioral reality monitoring post scanning, consistent with the earlier finding that increased cooperation between the FPN and DMN was associated with more rapid and accurate reality monitoring (Fornito et al., 2012).

Despite these changes in mPFC activity and the wider network rsFC associated with reality monitoring, our findings did not provide conclusive support for our prediction that active neurofeedback training targeting the mPFC would result in improved recollection of the source of self-generated information. In particular, the interaction term for the recognition of source of Imagined information compared with recognition memory, between group (Active vs. Sham neurofeedback) and session (pre and post scanning) fell short of a significant alpha value of .05, and there were no significant correlations in either group between the change in peak voxel signal and reality monitoring accuracy for Imagined items post scanning. However, the direction and effect size of the change in reality monitoring accuracy (i.e. post > pre) in the Active group, together with the associated changes in rsFC is consistent with the suggestion of increased cooperation between the DMN and FPN to support enhanced internally focused attention. Furthermore, while we did not detect significant effects on our behavioral measure, previous fMRI neurofeedback studies with similar samples have reported significant behavioral effects (e.g. Pamplona et al., 2020; Sherwood et al., 2016; Zhang et al., 2013a, 2013b). As such, a replication study may be of benefit in establishing whether a statistically significant reality monitoring behavioral effect is associated with fMRI neurofeedback training to the mPFC.

## 5 Conclusions

We have shown that healthy participants receiving Active neurofeedback were able to successfully self-regulate activity within the mPFC, which was associated with altered functional connectivity across regions and networks that may support reality monitoring performance. However, these activity and connectivity changes brought about by active neurofeedback training did not track with a clear improvement in accuracy for the recognition of the source of self-generated information and a replication study in a larger sample is proposed. It would also be interesting to extend the study to include a sample of patients with schizophrenia who experience hallucinations to explore whether improved reality monitoring and enhanced rsFC associated with neurofeedback to mPFC could reduce the intensity or frequency of hallucinatory experiences.

## CRediT authorship contribution statement

**J.R. Garrison:** Conceptualization, Data curation, Formal analysis, Writing – original draft. **F. Saviola:** Formal analysis, Writing – original draft. **E. Morgenroth:** Data curation. **H. Barker:** Data curation. **M. Lührs:** Conceptualization. **J.S. Simons:** Conceptualization, Writing – original draft. **C. Fernyhough:** Conceptualization, Writing – original draft. **P. Allen:** Project administration, Investigation.

## Declaration of Competing Interest

None.

## Appendix Supplementary materials

Image, application 1

## References

[bib0001] Abou Elseoud A., Littow H., Remes J., Starck T., Nikkinen J., Nissilä J., Timonen M., Tervonen O., Kiviniemi V. (2011). Group-ICA model order highlights patterns of functional brain connectivity. Front. Syst. Neurosci..

[bib0002] Abou-Elseoud A., Starck T., Remes J., Nikkinen J., Tervonen O., Kiviniemi V. (2010). The effect of model order selection in group PICA. Hum. Brain Mapp..

[bib0003] Bauer C.C.C., Okano K., Gosh S.S., Lee Y.J., Melero H., de los Angeles C., Nestor P.G., del Re E.C., Northoff G., Niznikiewicz M.A., Whitfield-Gabrieli S. (2020). Real-time fMRI neurofeedback reduces auditory hallucinations and modulates resting state connectivity of involved brain regions: part 2: default mode network -preliminary evidence. Psychiatry Res..

[bib0004] Beckmann C., Mackay C., Filippini N., Smith S. (2009). Group comparison of resting-state FMRI data using multi-subject ICA and dual regression. Neuroimage.

[bib0005] Bentall R.P., Slade P.D. (1985). Reliability of a scale measuring disposition towards hallucination: a brief report. Personal. Individ. Differ..

[bib0006] Brookwell M.L., Bentall R.P., Varese F. (2013). Externalizing biases and hallucinations in source-monitoring, self-monitoring and signal detection studies: a meta-analytic review. Psychol. Med..

[bib0007] Brunelin J., Combris M., Poulet E., Kallel L., D'Amato T., Dalery J., Saoud M. (2006). Source monitoring deficits in hallucinating compared to non-hallucinating patients with schizophrenia. Eur. Psychiatry.

[bib0008] Buda M., Fornito A., Bergstrom Z.M., Simons J.S. (2011). A specific brain structural basis for individual differences in reality monitoring. J. Neurosci..

[bib0009] Calhoun V., Pearlson G., Adali T. (2004). Independent component analysis applied to fMRI data: a generative model for validating results. J. VLSI Signal Process..

[bib0010] Cohen Kadosh K., Luo Q., de Burca C., Sokunbi M.O., Feng J., Linden D.E.J., Lau J.Y.F. (2016). Using real-time fMRI to influence effective connectivity in the developing emotion regulation network. Neuroimage.

[bib0011] Davey C.G., Pujol J., Harrison B.J. (2016). Mapping the self in the brain's default mode network. Neuroimage.

[bib0012] deCharms R.C., Maeda F., Glover G.H., Ludlow D., Pauly J.M., Soneji D., Gabrieli J.D.E., Mackey S.C. (2005). Control over brain activation and pain learned by using real-time functional MRI. Proc. Natl. Acad. Sci..

[bib0013] Fornito A., Harrison B.J., Zalesky A., Simons J.S. (2012). Competitive and cooperative dynamics of large-scale brain functional networks supporting recollection. Proc. Natl. Acad. Sci..

[bib0014] Fox M.D., Snyder A.Z., Vincent J.L., Corbetta M., Raichle M.E. (2005). The human brain is intrinsically organized into dynamic, anticorrelated functional networks. Proc. Natl. Acad. Sci..

[bib0015] Frith C.D., Done D.J. (1988). Towards a neuropsychology of schizophrenia. Br. J. Psychiatry.

[bib0016] Frith U., Frith C.D. (2003). Development and neurophysiology of mentalizing. Philos. Trans. R. Soc. Lond. Ser. B Biol. Sci..

[bib0017] Garrison J.R., Fernandez-Egea E., Zaman R., Agius M., Simons J.S. (2017). Reality monitoring impairment in schizophrenia reflects specific prefrontal cortex dysfunction. NeuroImage Clin..

[bib0018] Garrison J.R., Fernyhough C., McCarthy-Jones S., Haggard M., Simons J.S., The Australian Schizophrenia Research Bank (2015). Paracingulate sulcus morphology is associated with hallucinations in the human brain. Nat. Commun..

[bib0019] Garrison J.R., Moseley P., Alderson-Day B., Smailes D., Fernyhough C., Simons J.S. (2017). Testing continuum models of psychosis: no reduction in source monitoring ability in healthy individuals prone to auditory hallucinations. Cortex.

[bib0020] Garrison K.A., Scheinost D., Worhunsky P.D., Elwafi H.M., Thornhill T.A., Thompson E., Saron C., Desbordes G., Kober H., Hampson M., Gray J.R., Constable R.T., Papademetris X., Brewer J.A. (2013). Real-time fMRI links subjective experience with brain activity during focused attention. Neuroimage.

[bib0021] Golland Y., Golland P., Bentin S., Malach R. (2008). Data-driven clustering reveals a fundamental subdivision of the human cortex into two global systems. Neuropsychologia.

[bib0022] Harrison B.J., Pujol J., Lopez-Sola M., Hernandez-Ribas R., Deus J., Ortiz H., Soriano-Mas C., Yucel M., Pantelis C., Cardoner N. (2008). Consistency and functional specialization in the default mode brain network. Proc. Natl. Acad. Sci..

[bib0023] Hugdahl K., Kazimierczak K., Beresniewicz J., Kompus K., Westerhausen R., Ersland L., Gr R. (2019). Dynamic up- and down-regulation of the default (DMN) and extrinsic (EMN) mode networks during alternating task-on and task-off periods. PLoS ONE.

[bib0024] Humpston C., Garrison J., Orlov N., Aleman A., Jardri R., Fernyhough C., Allen P. (2020). Real-time functional magnetic resonance imaging neurofeedback for the relief of distressing auditory-verbal hallucinations: methodological and empirical advances. Schizophrenia Bulletin.

[bib0025] Jenkinson M., Bannister P., Brady M., Smith S. (2002). Improved optimization for the robust and accurate linear registration and motion correction of brain images. Neuroimage.

[bib0026] Jenkinson M., Beckmann C.F., Behrens T.E.J., Woolrich M.W., Smith S.M. (2012). FSL. NeuroImage.

[bib0027] Johnson M.K., Raye C.L. (1981). Reality monitoring. Psychol. Rev..

[bib0028] Li Y.O., Adalı T., Calhoun V.D. (2007). Estimating the number of independent components for functional magnetic resonance imaging data. Hum. Brain Mapp..

[bib0029] Linden D.E.J., Habes I., Johnston S.J., Linden S., Tatineni R., Subramanian L., Sorger B., Healy D., Goebel R. (2012). Real-time self-regulation of emotion networks in patients with depression. PLoS ONE.

[bib0030] Marek S., Dosenbach N. (2018). The frontoparietal network: function, electrophysiology, and importance of individual precision mapping. Dialogues Clin. Neurosci..

[bib0031] Mason O., Claridge G., Jackson M. (1995). New scales for the assessment of schizotypy. Personal. Individ. Differ..

[bib0032] Mason O., Linney Y., Claridge G. (2005). Short scales for measuring schizotypy. Schizophr. Res..

[bib0033] Mathiak K.A., Alawi E.M., Koush Y., Dyck M., Cordes J.S., Gaber T.J., Zepf F.D., Palomero-Gallagher N., Sarkheil P., Bergert S., Zvyagintsev M., Mathiak K. (2015). Social reward improves the voluntary control over localized brain activity in fMRI-based neurofeedback training. Front. Behav. Neurosci..

[bib0034] Metzak P.D., Lavigne K.M., Woodward T.S. (2015). Functional brain networks involved in reality monitoring. Neuropsychologia.

[bib0035] Morgenroth E., Orlov N., Lythgoe D.J., Stone J.M., Barker H., Munro J., Eysenck M., Allen P. (2019). Altered relationship between prefrontal glutamate and activation during cognitive control in people with high trait anxiety. Cortex.

[bib0036] Morrison A.P., Wells A., Nothard S. (2000). Cognitive factors in predisposition to auditory and visual hallucinations. Br. J. Clin. Psychol..

[bib0037] Nickerson L.D., Smith S.M., Öngür D., Beckmann C.F. (2017). Using dual regression to investigate network shape and amplitude in functional connectivity analyses. Front. Neurosci..

[bib0038] Orlov N.D., Giampietro V., O’Daly O., Lam S.L., Barker G.J., Rubia K., McGuire P., Shergill S.S., Allen P. (2018). Real-time fMRI neurofeedback to down-regulate superior temporal gyrus activity in patients with schizophrenia and auditory hallucinations: a proof-of-concept study. Transl. Psychiatry.

[bib0039] Pamplona G.S.P., Heldner J., Langner R., Koush Y., Michels L., Ionta S., Scharnowski F., Salmon C.E.G. (2020). Network-based fMRI-neurofeedback training of sustained attention. Neuroimage.

[bib0040] Qin P., Northoff G. (2011). How is our self related to midline regions and the default-mode network?. Neuroimage.

[bib0041] Raichle M.E. (2015). The brain's default mode network. Annu. Rev. Neurosci..

[bib0042] Rollins C.P.E., Garrison J.R., Arribas M., Seyedsalehi A., Li Z., Chan R.C.K., Yang J., Wang D., Liò P., Yan C., Yi Z., Cachia A., Upthegrove R., Deakin B., Simons J.S., Murray G.K., Suckling J. (2020). Evidence in cortical folding patterns for prenatal predispositions to hallucinations in schizophrenia. Transl. Psychiatry.

[bib0044] Sherwood M.S., Kane J.H., Weisend M.P., Parker J.G. (2016). Enhanced control of dorsolateral prefrontal cortex neurophysiology with real-time functional magnetic resonance imaging (rt-fMRI) neurofeedback training and working memory practice. Neuroimage.

[bib0045] Shulman G.L., Corbetta M., Buckner R.L., Fiez J.A., Miezin F.M., Raichle M.E., Petersen S.E. (1997). Common blood flow changes across visual tasks: I. increases in subcortical structures and cerebellum but not in nonvisual cortex. J. Cogn. Neurosci..

[bib0046] Simons J., Henson R., Gilbert S., Fletcher P. (2008). Separable forms of reality monitoring supported by anterior prefrontal cortex. J. Cogn. Neurosci..

[bib0047] Simons J.S., Davis S.W., Gilbert S.J., Frith C.D., Burgess P.W. (2006). Discriminating imagined from perceived information engages brain areas implicated in schizophrenia. Neuroimage.

[bib0048] Simons J.S., Garrison J.R., Johnson M.K. (2017). Brain mechanisms of reality monitoring. Trends Cogn. Sci..

[bib0049] Smith S., Nichols T. (2009). Threshold-free cluster enhancement: addressing problems of smoothing, threshold dependence and localisation in cluster inference. Neuroimage.

[bib0050] Smith S.M. (2002). Fast robust automated brain extraction. Hum. Brain Mapp..

[bib0051] Spreng R.N., Sepulcre J., Turner G.R., Stevens W.D., Schacter D.L. (2013). Intrinsic architecture underlying the relations among the default, dorsal attention, and frontoparietal control networks of the human brain. J. Cogn. Neurosci..

[bib0052] Subramaniam K., Kothare H., Hinkley L.B., Tarapore P., Nagarajan S.S. (2020). Establishing a causal role for medial prefrontal cortex in reality monitoring. Front. Hum. Neurosci..

[bib0053] Thibault R.T., MacPherson A., Lifshitz M., Roth R.R., Raz A. (2018). Neurofeedback with fMRI: a critical systematic review. Neuroimage.

[bib0054] Turner M.S., Simons J.S., Gilbert S.J., Frith C.D., Burgess P.W. (2008). Distinct roles for lateral and medial rostral prefrontal cortex in source monitoring of perceived and imagined events. Neuropsychologia.

[bib0055] Uddin L.Q., Yeo B.T.T., Spreng R.N. (2019). Towards a universal taxonomy of macro-scale functional human brain networks. Brain Topogr..

[bib0056] van der Meer L., Costafreda S., Aleman A., David A.S. (2010). Self-reflection and the brain: a theoretical review and meta-analysis of neuroimaging studies with implications for schizophrenia. Neurosci. Biobehav. Rev..

[bib0057] Vinogradov S., Luks T.L., Schulman B.J., Simpson G.V. (2008). Deficit in a neural correlate of reality monitoring in schizophrenia patients. Cereb. Cortex.

[bib0058] Waters F.A.V., Maybery M.T., Badcock J.C., Michie P.T. (2004). Context memory and binding in schizophrenia. Schizophr. Res..

[bib0059] Whalley H.C., Simonotto E., Flett S., Marshall I., Ebmeier K.P., Owens D.G.C., Goddard N.H., Johnstone E.C., Lawrie S.M. (2004). fMRI correlates of state and trait effects in subjects at genetically enhanced risk of schizophrenia. Brain.

[bib0060] Woodward T.S., Menon M., Jardri R., Pins D., Cachia A., Thomas P. (2014). The Neuroscience of Hallucinations.

[bib0061] Worsley K.J., Friston K.J. (1995). Analysis of fMRI time-series revisited-again. Neuroimage.

[bib0062] Yeo T.B.T., Krienen F.M., Sepulcre J., Sabuncu M.R., Lashkari D., Hollinshead M., Roffman J.L., Smoller J.W., Zöllei L., Polimeni J.R., Fischl B., Liu H., Buckner R.L. (2011). The organization of the human cerebral cortex estimated by intrinsic functional connectivity. J. Neurophysiol..

[bib0063] Young K.D., Zotev V., Phillips R., Misaki M., Yuan H., Drevets W.C., Bodurka J. (2014). Real-time fMRI neurofeedback training of amygdala activity in patients with major depressive disorder. PLoS ONE.

[bib0064] Zhang G., Yao L., Zhang H., Long Z., Zhao X. (2013). Improved working memory performance through self-regulation of dorsal lateral prefrontal cortex activation using real-time fMRI. PLoS ONE.

[bib0065] Zhang G., Zhang H., Li X., Zhao X., Yao L., Long Z. (2013). Functional alteration of the DMN by learned regulation of the PCC using real-time fMRI. IEEE Trans. Neural Syst. Rehabil. Eng..

[bib0066] Zilverstand A., Sorger B., Slaats-Willemse D., Kan C.C., Goebel R., Buitelaar J.K. (2017). fMRI Neurofeedback training for increasing anterior cingulate cortex activation in adult attention deficit hyperactivity disorder. An exploratory randomized, single-blinded study. PLoS ONE.

