# Supplementary material for: Modulating medial prefrontal cortex activity using real-time fMRI neurofeedback: Effects on reality monitoring performance and associated functional connectivity
Source: Neuroimage. 2021 Dec 15;245:118640. doi: 10.1016/j.neuroimage.2021.118640 (PMC8752965; doi:10.1016/j.neuroimage.2021.118640)
Supplement: Supplementary file 1 [file mmc1.docx]

**SUPPLEMENTARY MATERIALS**


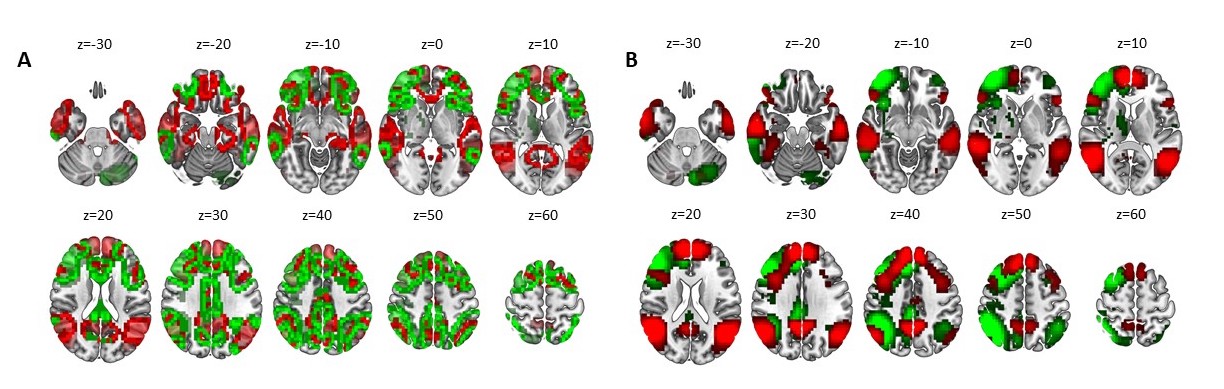


***Figure S1.*** *Functional Networks and chosen Independent Components. (A) Standard DMN (red) and FPN (green) from the 7-network parcellation of the human cortex by Yeo et al. (2011). (B) The two Independent Components selected for analysis from the resting state data. These show a high level of overlap with the standard networks. Pearson’s correlation coefficients between the standard network and its associated Independent Component: DMN r = .570; FPN r = .537*


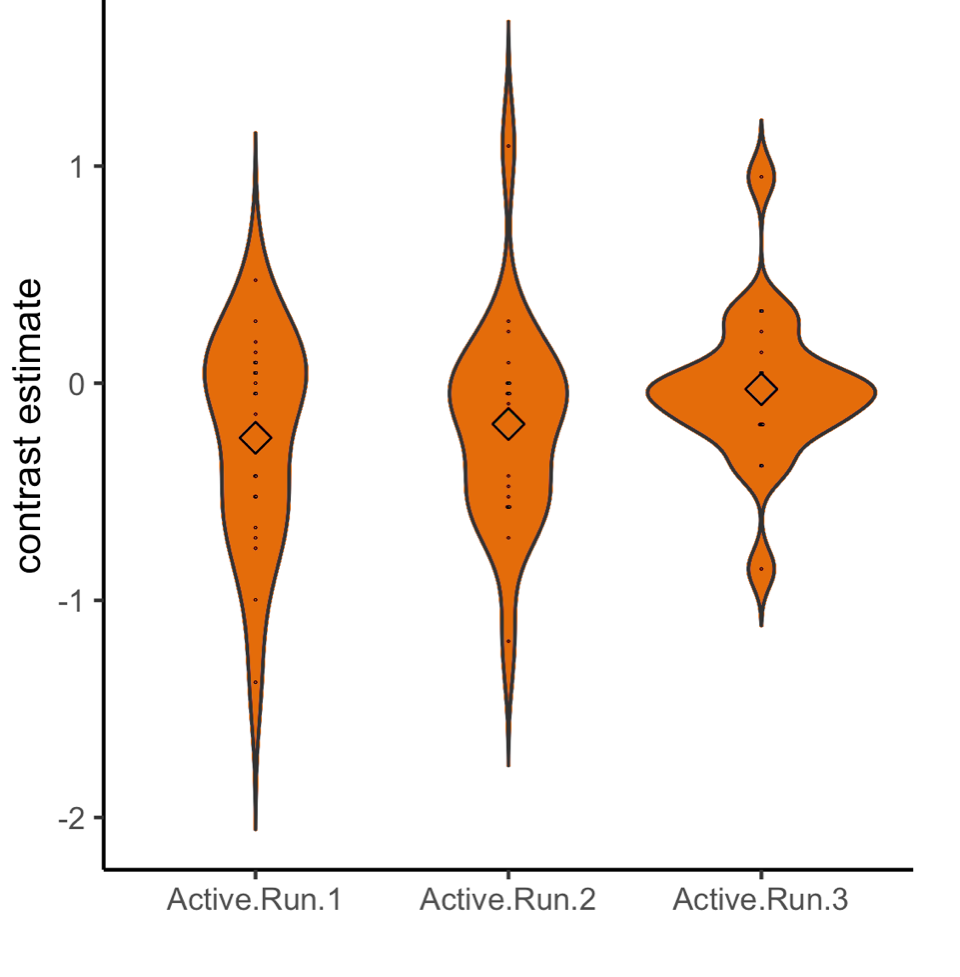

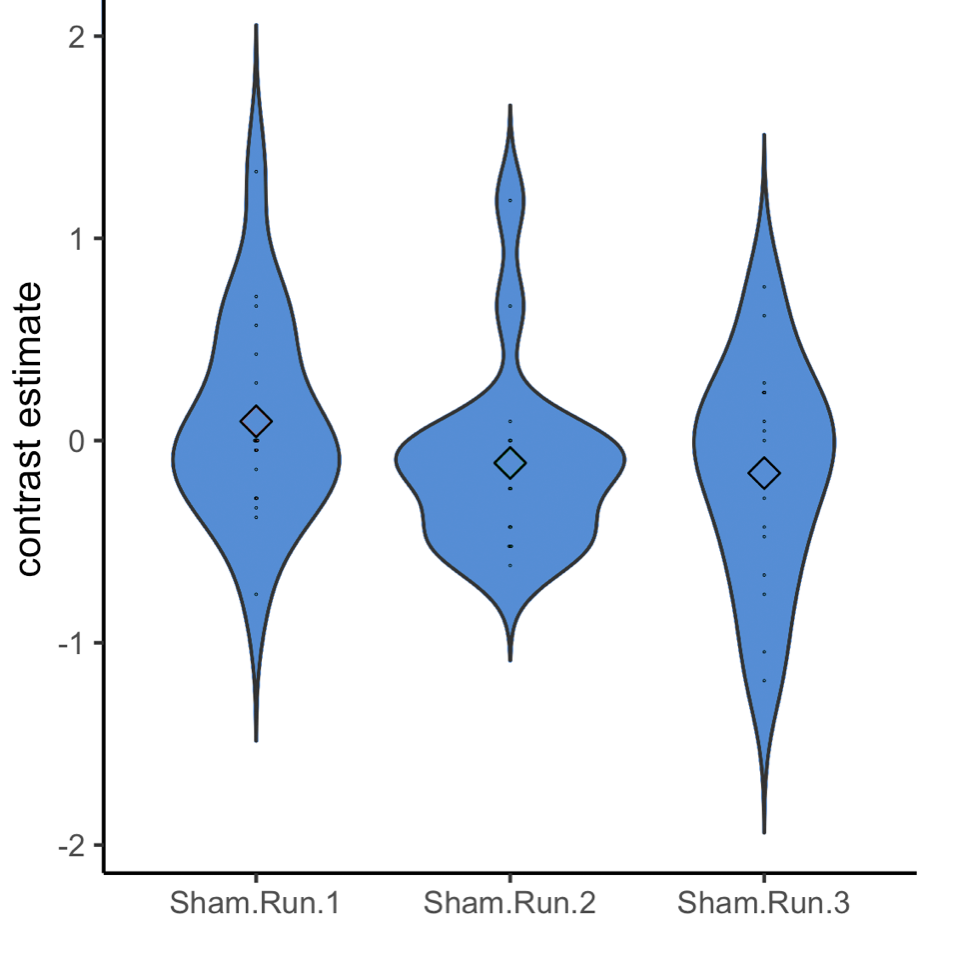


***Figure S2****. Violin plots showing distribution of the fMRI contrast estimate from the peak mPFC voxel [8, 48, -4] across the three scanning runs for participants in the Active (orange) and Sham (blue) groups (see Figure 3). Diamonds indicate group means.*


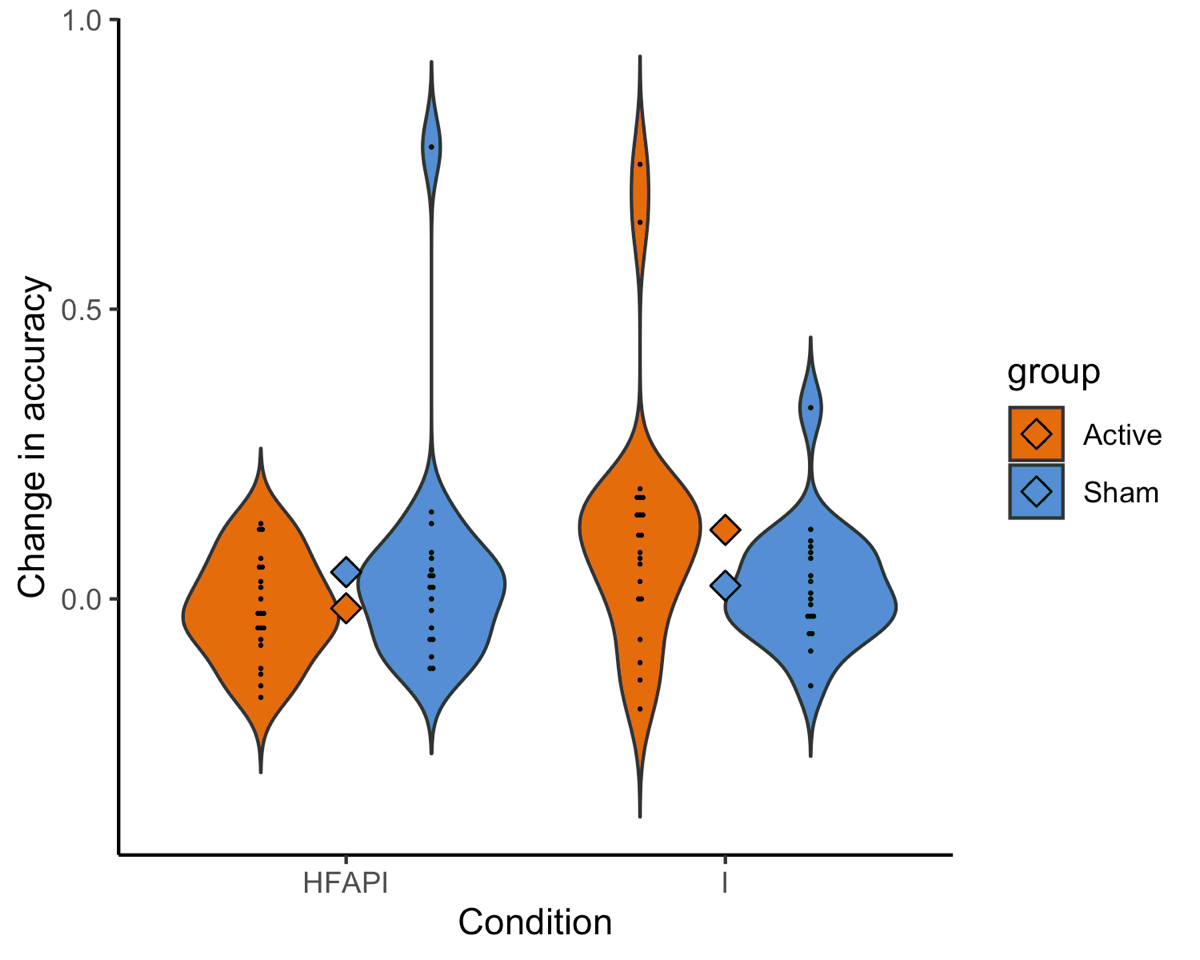


Recognition Memory

Imagined

***Figure S3****. Violin plots showing distribution of the change in accuracy (post > pre fMRI neurofeedback training) for the reality monitoring for Imagined items and Recognition Memory conditions for participants in the Active (orange) and Sham (blue) groups. The colored diamonds indicate group means. Note: the plot highlights three outliers for the Imagined condition and one for the Recognition memory condition (four participants in total). There was no major effect of removing these participants on the results of the group x session x condition mixed ANOVA analysis presented in the Results section 3.2: there were no significant mains effects or two way interactions, with a reduced trend effect in the three way interaction of group x session x memory condition, F(33,1) = 1.964, p = .170, η_p_^2^ =.056.*

***
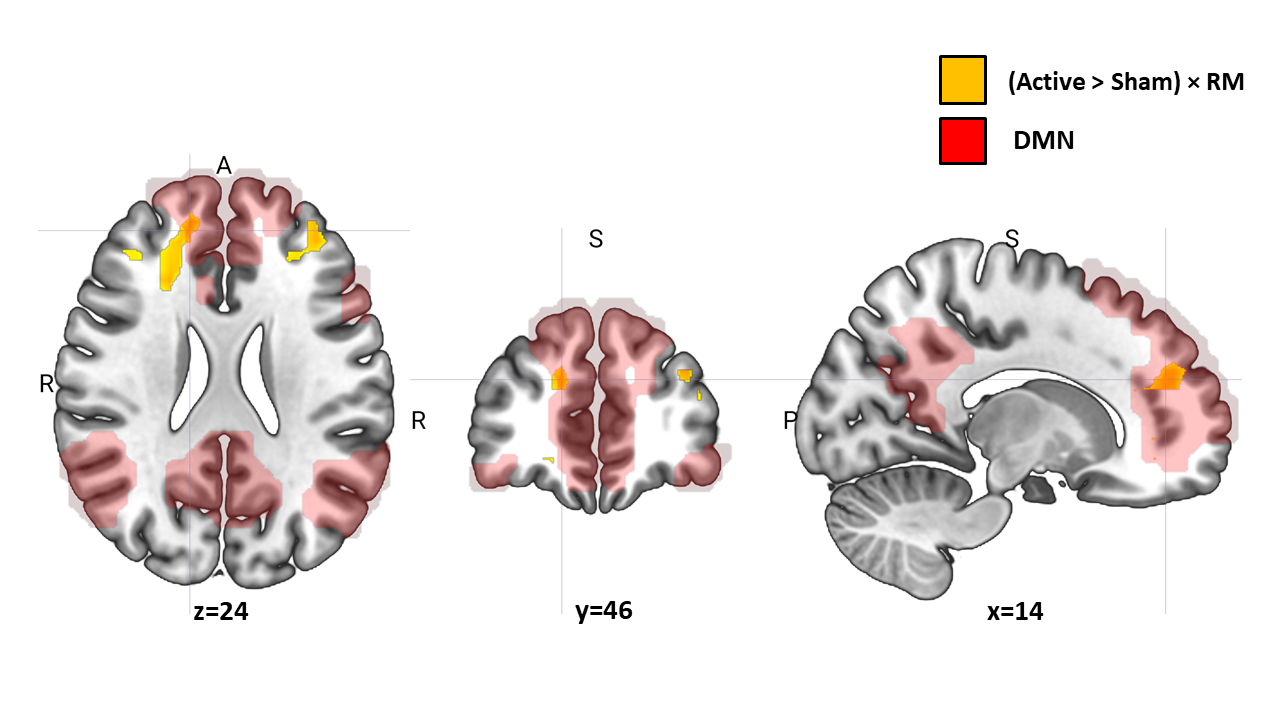
***

***Figure S4****. ICA-based rsFC results for the Active > Sham group interaction with change in reality monitoring accuracy for Imagined items within the FPN (yellow), shown against an overlay of the standard DMN (red, Yeo et al., 2011). Significant results are visualized for p-value < .05 FWE voxel-wise corrected and p-value < .05 for multiple Independent Components testing. The slices are centered on the FPN paracingulate gyrus cluster (Table 3) to highlight possible cooperation between the FPN and DMN regions associated with increased attention to internally focused tasks.*

***
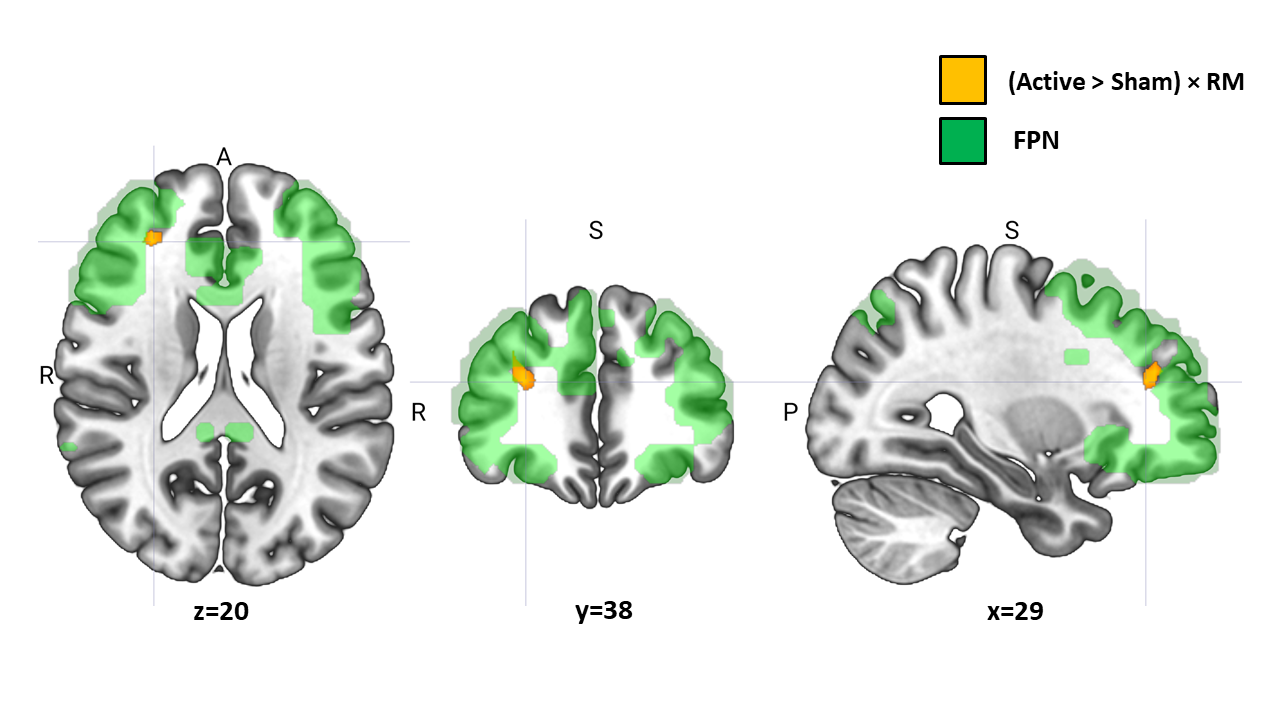
***

***Figure S5****. ICA-based rsFC results for the Active > Sham group interaction with change in reality monitoring accuracy for Imagined items within the DMN (yellow), shown against an overlay of the standard FPN (green; Yeo et al., 2011). Significant results are visualized for p-value < .05 FWE voxel-wise corrected and p-value < .05 for multiple Independent Components testing. The slices are centered on the DMN middle frontal gyrus cluster (Table 2) to highlight possible cooperation between the FPN and DMN regions associated with increased attention to internally focused tasks.*

******

***Figure S6****. ICA-based rsFC scatter plots for the interaction between the change in reality monitoring accuracy for Imagined items within the fronto-parietal and default mode networks for participants in the Active (orange) compared to Sham (light blue) groups. X-axis = rsFC parameter estimates in significant voxels for the interaction comparison (see Tables 2 and 3), Y-axis = post scanning > pre scanning change in reality monitoring accuracy for Imagined items*

******

***Figure S7****. CRED-nf Checklist for best practice in reporting fMRI neurofeedback studies*

*(Ros et al., 2020.* Consensus on the reporting and experimental design of clinical and cognitive-behavioral neurofeedback studies (CRED-nf checklist). Brain 143, 1674–1685.)
